# A Study of the Correlation between the Bulkiness of *peri*-Substituents and the Distortion of a Naphthalene Ring

**DOI:** 10.3390/molecules28145343

**Published:** 2023-07-11

**Authors:** Annisa Indah Reza, Kento Iwai, Nagatoshi Nishiwaki

**Affiliations:** 1 School of Engineering Science, Kochi University of Technology, Tosayamada, Kami, Kochi 782-8502, Japan; 256001e@gs.kochi-tech.ac.jp (A.I.R.); iwai.kento@kochi-tech.ac.jp (K.I.); 2 Research Center for Molecular Design, Kochi University of Technology, Tosayamada, Kami, Kochi 782-8502, Japan

**Keywords:** steric repulsion, *peri*-substituents, non-electronic activation, naphthalene, negative hyperconjugation

## Abstract

A systematic study on the distortion of a naphthalene ring was performed using steric repulsion between *peri*-substituents at the 1- and 8-positions. The introduction of bromo groups into the methyl groups of the 1,8-dimethylnaphthalene enhanced the steric repulsion to distort the naphthalene ring. X-ray crystallography revealed that 1,8-bis(bromomethyl)naphthalene had a vertical distortion with a 11.0° dihedral angle (α) between *peri*-substituents which disturbed the coplanarity of the naphthalene ring. On the other hand, the dihedral angle of 1,8-bis(dibromomethyl)naphthalene was smaller (α = 8.3°) despite the bulkier substituents. In this case, horizontal distortion of the naphthalene ring increased. These distortions should non-electronically activate the naphthalene framework. In order to evaluate their reactivity, nitration and hydrogenation were carried out; however, the 1,8-bis(dibromomethyl)naphthalene was intact under the employed conditions. A DFT calculation suggested that the inertness of the 1,8-bis(dibromomethyl)naphthalene is presumably due to the negative hyperconjugation of the (dibromo)methyl group.

## 1. Introduction

Aromatic compounds have played essential roles in the material and pharmaceutical sciences. Accordingly, numerous methods for modifying aromatic rings have been developed. The most frequently used reactions are cross-coupling reactions, reactions using benzynes, and electrophilic substitution. In the former two cases, the introduction of a good leaving group such as a halide or sulfonate is important for initiating the reactions, that is, reactive carbon–halogen or carbon–sulfonate bonds facilitate chemical modification. In the latter case, the substituent effect is crucial for promoting the reaction. Although the introduction of an electron-donating or electron-withdrawing group is the most general activation method for an aromatic ring, the reaction site will be restricted because of their directing properties. Thus, while an enormous number of reactions were reported, the development of another type of activation protocol is a relatively unexplored field.

**Scheme 1:**
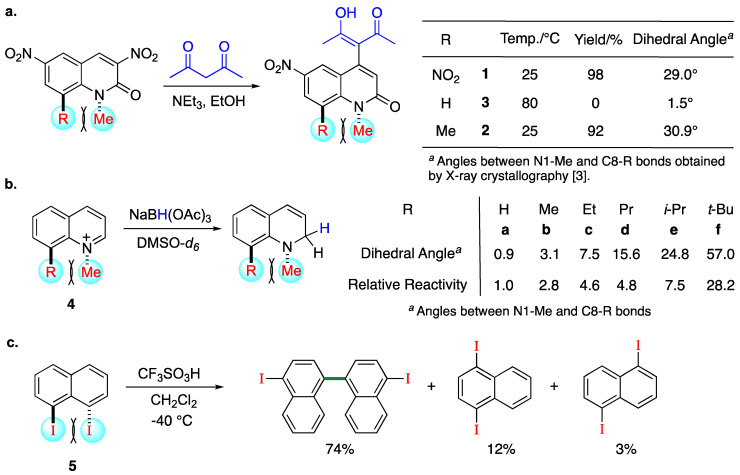
(**a**) Non-electronic activation of 8-substituted MeQone derivatives; (**b**) comparing reactivities of 8-alkylated 1-methylquinolinium salts; (**c**) homocoupling and halogen dance reaction of 1,8-diiodonaphthalene.

In our previous work, we studied the functionalization of the 1-methyl-2-quinolone framework. During the study, we found that 1-methyl-3,6,8-trinitro-2-quinolone (1) exhibits unusually high reactivities. When 1 was allowed to react with 2,4-pentanedione, *cine*-substitution efficiently proceeded on the pyridone moiety at room temperature. Furthermore, 3,6-dinitroquinolone 3 exhibited similar reactivity to 1 even though the 8-substituent was replaced by an electron-donating methyl group. In contrast, no reaction occurred for 8-unsubstituted 3,6-dinitiroquinolone 2, even under heating conditions. Since the 8-position is distant from the reaction site and this reaction was not influenced by the electronic property of the 8-substituent, the steric effect of the substituent was concluded to be crucial. Indeed, X-ray crystallography of 1 showed that the dihedral angle between the N1–Me and C8–NO_2_ bonds was 25°. Based on these experimental results, we speculated that steric repulsion between the 1-methyl group and 8-substituent distorts the quinolone framework to decrease the aromaticity, and the pyridine moiety serves as the activated nitroalkene (Scheme 1a) [1]. Namely, it is non-electrical activation of an aromatic ring. As an associated study, the aromaticity and reactivity of distorted aromatic compounds were reported [2,3,4].

Inspired by these results, a systematic study on the correlation between steric repulsion and reactivity was performed using 1-methyl-8-alkylquinolinium salts 4a–f (Scheme 1b) [5]. As the 8-substituent becomes bulkier, the dihedral angles between the N1–Me and C8–R bonds become larger, inducing distortion of the quinoline ring. Accordingly, the highly distorted quinolinium salts 4e and 4f underwent reactions with sodium tri(acetoxy)borohydride efficiently (Scheme 1b). More recently, we also reported the transformation of 1,8-diiodonaphthalene via non-electronical activation. The 1,8-diiodonaphthalene showed various reactivities to undergo a homocoupling reaction, leading to binaphthyl and the rearrangement of the iodo group (Scheme 1c) [6].

As shown in Scheme 1, the steric repulsion between *peri*-substituents effectively activates fused aromatic compounds such as quinoline and naphthalene. On the other hand, to the best of our knowledge, a systematic study on the relationship between the bulkiness of *peri*-substituents and the distortion of the naphthalene ring has not been studied. Nevertheless, there are several studies on the interactions between *peri*-substituents from various perspectives, such as intramolecular coordination [7,8,9,10,11,12], chelation assembly [13,14], and mechanophores [15]. In the present work, 1,8-dimethylnaphthalene **7** was chosen as a model compound, and the bulkiness of the *peri*-substituents was increased via the sequential bromination of the methyl groups. Moreover, we investigated the reactivity of the distorted naphthalene ring using nitration and hydrogenation reactions.

## 2. Results and Discussion

### 2.1. Synthesis of 1,8-Disubstituted Naphthalenes

**Scheme 2:**
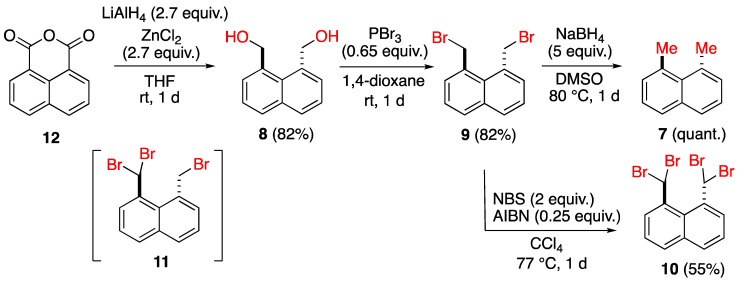
Preparation of 1,8-disubstituted naphthalenes **7**–**10**.

According to the works [16,17], 1,8-disubstituted naphthalenes **7**–**10** were synthesized from 1,8-naphthalic anhydride **12** (Supplementary Materials). Namely, According to the works, 1,8-disubstituted naphthalenes **7**–**10** were synthesized from 1,8-naphthalic anhydride 122 (Scheme 2). The reduction of **12** with lithium aluminum hydride in the presence of zinc chloride afforded bis(hydroxymethyl) derivative **8**. The hydroxy groups were efficiently converted into bromomethyl groups upon heating with phosphorous tribromide, which led to bis(bromomethyl) derivative **9**. The subsequent reduction of **9** with sodium borohydride qualitatively yielded 1,8-dimethylnaphthalene **7** [18]. Bromination at the benzyl position of **9** by *N*-bromosuccinimide (NBS) furnished bis(dibromomethyl) derivative **10**; however, an intermediately expected product wuch as tribrominated product **11** was not detected, even when an equimolar amount of NBS was used. Although the direct synthesis of **11** from **7** or **8** was also attempted using NBS, **11** remained undetectable [19].

### 2.2. Crystal Structure Study

**Figure 1:**
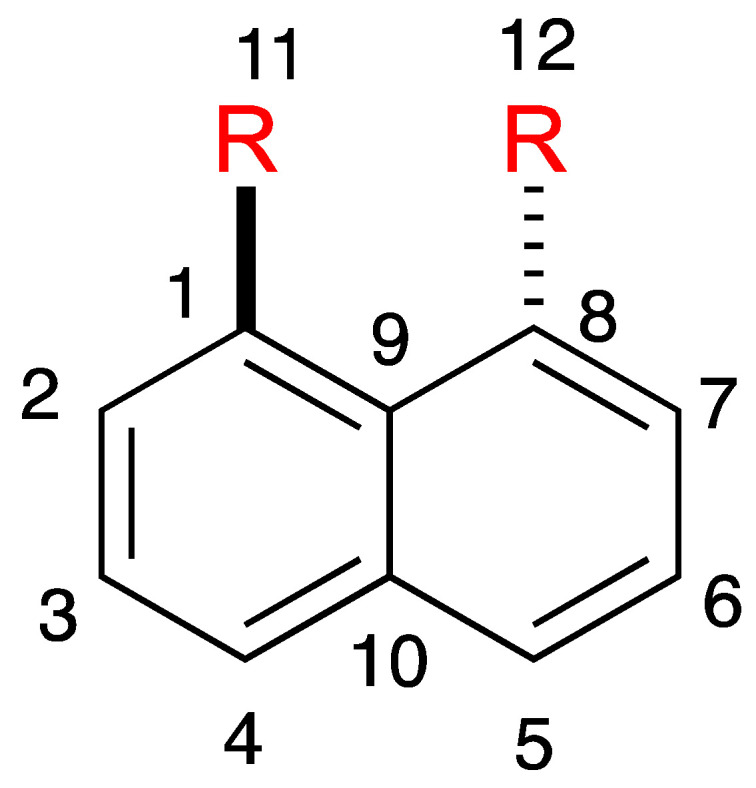
The order of atoms in the crystal structures of the naphthalene derivatives.

**Figure 2:**
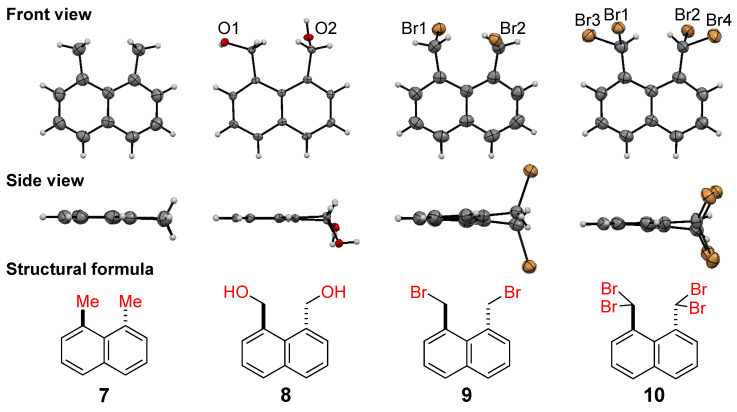
ORTEP drawing of naphthalene derivatives **7**–**10** with displacement ellipsoids at the 50% probability level.

Since four kinds of 1,8-disubstituted naphthalenes, **7**–**10**, were in hand, their single crystals were subjected to X-ray crystallography. The order of atoms (Figure 1) and ORTEP views from the top and side directions are shown in Figure 2, respectively. Selected crystal parameters for naphthalene derivatives are also presented in Table 1.

The distortion of the naphthalene ring was evaluated using three parameters, namely, (a) vertical distortion, (b) horizontal distortion, and (c) bond length and interatom distance [11]. In this section, these parameters are compared and discussed for naphthalenes **7**–**10**.

#### 2.2.1. Vertical Distortion

The vertical distortion of the naphthalene framework was estimated via a comparison of the three dihedral angles α–γ (Table 2). Referring to our previous work [4,5], we initially assessed the vertical distortion using a dihedral angle α between the C1–C11 and C8–C12 bonds. In the case of dimethylnaphthalene **7**, the dihedral angle α is 0.7°, indicating that coplanarity remains. Introducing a hydroxy group to the methyl groups distorted the naphthalene ring to some extent. More importantly, the larger bromo groups distorted the naphthalene ring to a greater degree, as can be seen with the dihedral angle α reaching 11.0°. Interestingly, bis(dibromomethyl)naphthalene **10** is less distorted than **9**, even though two bromine atoms were introduced to the methyl group. 

As other parameters to evaluate the vertical distortion, the dihedral angles β (between the C1–C9 and C4–C10 bonds) and γ (between the C1–C9 and C5–C10 bonds) were compared [20]. While the dihedral angles β and γ of dimethylnaphthalene 7 are quite small (β = 0.5° and γ = 0.1°), the values of other naphthalenes **8**–**10** increase depending on the bulkiness of the substituents. Meanwhile, the dihedral angles of **10** were not larger than those of **9**, which is the same tendency as α. Since the substitution of the bulkier dibromomethyl groups should induce more prominent ring distortion, an alternative parameter to evaluate the distortion was required.

#### 2.2.2. Horizontal Distortion

Bond angles around the C9 position are shown in Table 3. Since the sum of the three angles is 360°, the three atoms connected to the C9 carbon are located in the same plane; however, the angles are not equally divided. All compounds have wider angles δ (outside of the naphthalene ring) than the standard *sp^2^* bond angle (120°), while the other two angles ε and ζ (inside of the naphthalene ring) are narrower. This fact indicates that C1 and C8 were pushed apart via steric repulsion between the *peri*-substituents. Accordingly, the outside angle θ at the opposite side (C4–C10–C5 angle) became smaller. 

Notably, each compound exhibited similar bond angle tendencies regardless of the bulkiness of the substituents. This result suggests that the first distortion occurs horizontally, absorbing repulsive energy, when the *peri*-substituents become bulky.

#### 2.2.3. Bond Length and Interatom Distance

Steric repulsion between *peri*-substituents should affect not only bond angles but also bond distances and interatom distances. Some selected bond lengths and interatom distances are shown in Table 4 and Table 5, respectively. While the bond lengths of **7**–**9** resemble each other, a partial stretch of the inner bond length (C1–C9) of bis(dibromomethyl)naphthalene **10** was observed, which is about 0.035 Å longer than that of the other naphthalenes, **7**–**9**. On the other hand, the outer bond lengths (C1–C2, C2–C3, and C4–C10) of **10** are shorter than those of the other derivatives. This is probably due to the longer bond length, as the vertical dihedral angles α–γ in **10** are smaller than those of **9**. The interatom distances also indicate high degree of ring distortion in naphthalene **10**, that is, C1–C8 and C2–C9 were longer than others. On the other hand, the distance between C4 and C5 on the opposite side is shorter than those of other substrates, which is a result of a compression of the naphthalene ring. Since the C11–C12 distances are longer in naphthalenes **8**–**10** than in **7**, the *peri*-substituents part from each other to absorb repulsive energy, which causes horizontal and vertical distortions. 

#### 2.2.4. Mechanism of Distortion

**Figure 3:**
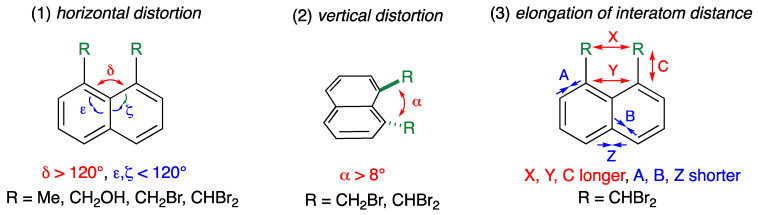
Three kinds of distortion of the naphthalene ring.

To summarize our discussions above, the ring distortion occurs in the following order: (1) horizontal distortion, (2) vertical distortion, and (3) the elongation of the bond and interatom distances as their bulkiness increases (Figure 3). In detail, when two substituents exist at the *peri*-positions, the outside ring angles around C9 δ become larger than the *sp*^2^ standard angle (120°), while the inside ring angles ε and ζ become smaller (horizontal distortion). During this distortion, coplanarity remained for compound **7**. A larger steric repulsion disturbs the coplanarity of the naphthalene ring (dihedral angles α–γ) upon the introduction of hydroxy or bromo groups into the methyl groups (vertical distortion). As the substituent becomes bulkier, interatom distances X and Y become longer, and the bond length C also elongates due to the steric repulsion between the *peri*-substituents (the elongation of bond and interatom distances). On the other hand, the bond lengths represented by A and B, in addition to the interatomic distance denoted by Z on the opposite side, experience reductions in length due to compression.

### 2.3. Evaluation of the Reactivity

In the last section, the distortion of naphthalene ring is discussed. According to the distorted structures, their reactivity should increase because of the decrease in aromaticity. Therefore, the reactivities of the 1,8-disubstituted naphthalenes **7**–**10** were evaluated to strengthen this hypothesis. For this purpose, nitration [21] and catalytic hydrogenation were employed as model reactions.

#### 2.3.1. Nitration Reaction

When 1,8-dimethylnaphthalene 7 was reacted with nitric acid in acetic anhydride, the nitration efficiently proceeded at room temperature to afford 2- and 4-nitrated products in 44% and 56% yields, respectively (Table 6, Entry 1, Supplementary Materials). However, brominated naphthalene 9 was less reactive, which led to a nitrated product with only a 12% yield (Entry 2, Supplementary Materials). Moreover, tetrabrominated naphthalene 10 was recovered intact under the conditions employed (Entry 3). These results contradicted our expectation, presumably due to the electronic effect of the bromomethyl groups in which the electron-withdrawing inductive effect diminishes the electron density of the naphthalene ring. 

^1^H NMR spectra showed that signals of ring protons shifted downfield as the bromo groups increased, indicating low electron densities on the naphthalene rings of 9 and 10. Since the electronic effects of bromomethyl groups are somewhat high, an electrophilic aromatic substitution such as nitration reaction is considered to be unsuitable for evaluating the non-electronic activation degrees of the naphthalenes.

#### 2.3.2. Hydrogenation Reaction

**Scheme 3:**
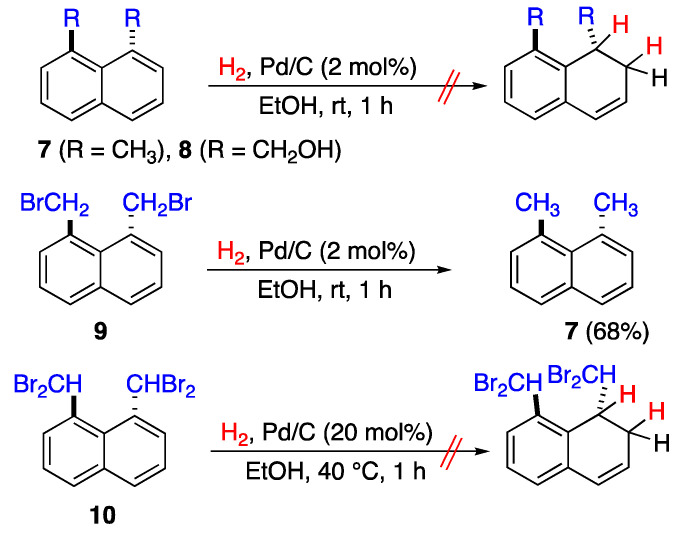
Hydrogenation of 1,8-disubstituted naphthalenes **7**–**10**.

As another approach, catalytic hydrogenation was chosen, which should be influenced less by the electronic effects of the substituents. The naphthalene derivatives were stirred under hydrogen with atmospheric pressure in the presence of a Pd/C catalyst in ethanol at room temperature for 1 h (Scheme 3). 

While naphthalenes **7**, **8**, and **10** did not react under the conditions, bromomethyl-substituted naphthalene **9** proceeded through the reaction to afford dimethylnaphthalene **7** in a 68% yield. Although the reaction occurred with substituents, the high level of reactivity of **9** is interesting to compare with the inertness of **10**.

#### 2.3.3. Another Effect of a Bromo Group

Among the *peri*-substituted naphthalenes **7**–**10**, only **9** exhibited a high degree of reactivity for hydrogenation. From the perspective of distortion, tetrabrominated naphthalene **10** is considered to be more reactive; however, **10** is, in fact, less reactive than **9**. One possible reason why the catalytic surface was not approachable during the hydrogenation reaction could be due to the steric hindrance caused by the dibromomethyl group. Nevertheless, another reason should be considered to explain the unusual reactivity of **9**.

To obtain insight, density functional theory (DFT) calculations for **7**, **9**, and **10** were performed. The calculated structural parameters were similar to the actual parameters observed via crystallography (Table 7). Characteristically, the bond lengths between the substituent and the ring carbons (C1–C11 and C8–C12) are shorter in the case of **9**, indicating these bonds possess double-bond properties. The carbon–bromine bonds (C11–Br1 and C12–Br2) of the brominated naphthalenes **9** and **10** were orthogonal to the naphthalene rings and elongated. On the other hand, the second carbon–bromine bonds (C11–Br3 and C12–Br4) in **10** are shorter than them. The HOMO levels were lowered as bromo groups were introduced (Table 8). These results indicate that the carbon–bromine bonds interacted with the π system of the naphthalene ring, and π electrons were delocalized to the anti-bonding orbital of the carbon–bromine bond (σ*), which facilitated the bond fission to demonstrate the high reactivity of **9**. However, the introduction of the second bromine increased the steric hindrance, which resulted in the inertness of **10**.

#### 2.3.4. NIC S Calculations

To estimate the aromaticity, NICS calculations were performed for naphthalenes **7**, **9**, and **10** (Table 9). The NICS(1) value is considered different on the front and back side of the ring plane because one of the carbon–bromine bonds is orthogonal to the naphthalene ring; thus, both calculations NICS(-1) and NICS(1) were performed. 

The NICS(0) values of bis(bromomethyl)naphthalene **9** and bis(dibromomethyl)naphthalene **10** were estimated to −9.9 and −9.3, respectively, which are lower fields than that of dimethylnaphthalene **7** (−9.0). In the case of NICS(1 or −1), a ghost atom (Bq) on the side with the orthogonal bromo group showed a higher field shift than that on another side. To avoid the electronic effect of the bromo group, NICS(1 or −1) values without an orthogonal bromo group (NICS(−1) for ring A and NICS(1) for ring B) were compared. As a result, **7** shows the greatest upfield shift among the naphthalenes. Although these results should indicate a higher degree of magnetic anisotropy of **7** than **9** and **10**, the electron-withdrawing effect of the bromo group, as mentioned in the last section, must be taken into account. At least a comparison of **10** with **9** shows an upfield shift via increasing ring distortion, which probably indicates the lesser degree of magnetic anisotropy of **10** than **9**.

## 3. Materials and Methods

All reagents were purchased from commercial sources and used without further purification. The progress of the reactions was monitored via TLC on silica gel 60 F254 on aluminum plates. ^1^H and ^13^C NMR spectra were recorded using a JEOL JMN-ECZ400S spectrometer (400 MHz and 100 MHz, respectively) in CDCl_3_, using TMS as an internal standard. The assignments of the ^13^C NMR were measured via DEPT experiments. The order of the aromatic protons of the naphthalenes was confirmed via 2D-NOESY. IR spectra were recorded with a JASCO FT/IR-4200 spectrometer equipped with an ATR detector. The melting points were recorded on an SRS-Optimelt automated melting point system. Diffraction data were collected at 103 K under a cold N_2_ gas stream on a Rigaku XtaLAB Synergy-S/Mo system (λ = 0.71073 Å (Mo-Kα)). The single crystals for each compound were obtained via recrystallization from CHCl_3_. High-resolution mass spectra (HRMS) values were obtained from a Bruker compact mass spectrometer set at an APCI-positive mode. The geometrical optimization was carried out via DFT calculation at the B3LYP/6-31g(d,p) level using the Gaussian 09 package.

**Preparation of 1,8-dimethylnaphthalene (7) [18]**

To a solution of 1,8-bis(bromomethyl)naphthalene (**9**) (1.6 g, 5 mmol) in dimethyl sulfoxide (20 mL), sodium borohydride (970 mg, 25 mmol) was added, and the resulting mixture was heated at 80 °C for 1 day. Water (20 mL) was added and stirred at room temperature for 1 h. The white precipitates were collected via filtration and purified via column chromatography on silica gel to afford 1,8-dimethylnaphthalene (**7**) (0.78 g, 5 mmol, quant., eluted by hexane/EtOAc = 9/1) as colorless needles. ^1^H NMR (400 MHz, CDCl_3_) δ 7.67 (d, *J* = 8.0 Hz, 2H), 7.31–7.23 (m, 4H), 2.94 (s, 6H).

**Preparation of 1,8-bis(hydroxymethyl)naphthalene (8) [18]**

To a suspension of lithium aluminum hydride (2.6 g, 68 mmol) in tetrahydrofuran (200 mL), zinc chloride (2.2 g, 68 mmol) was added at −20 °C. Then, 1,8-naphthalic anhydride (**1**) (5 g, 25 mmol) was slowly added, and the resulting mixture was stirred at room temperature for 1 d. After quenching the reaction with water (20 mL), the pH was adjusted to 4–5 with 1 M of hydrochloric acid. After the removal of THF and extraction with ethyl acetate (30 mL × 3), the organic layer was washed with brine (30 mL), dried over magnesium sulfate, and concentrated under reduced pressure. The residue was washed with diethyl ether (50 mL) to afford 1,8-bis(hydroxymethyl)naphthalene (**8**) (3.85 g, 20.5 mmol, 82%) as colorless needles. ^1^H NMR (400 MHz, CDCl_3_) δ 7.87 (dd, *J* = 8.2, 1.6 Hz, 2H), 7.56 (dd, *J* = 7.0, 1.6 Hz, 2H), 7.45 (dd, *J* = 8.2, 7.0 Hz, 2H), 5.30 (s, 4H).

**Preparation of 1,8-bis(bromomethyl)naphthalene (9) [18]**

To a solution of naphthalene **3** (3.0 g, 16 mmol) in 1,4-dioxane (30 mL), phosphorous tribromide (1 mL, 10.5 mmol) was slowly added, and the mixture was stirred at room temperature for 1 d. After the addition of water (10 mL), the mixture was stirred for 1 h, and colorless precipitates were collected via filtration and dried under vacuum to furnish 1,8-bis(bromomethyl)naphthalene (**9**) (4.0 g, 12.7 mmol, 82%). ^1^H NMR (400 MHz, CDCl_3_) δ 7.89 (dd, *J* = 8.2, 1.6 Hz, 2H), 7.63 (dd, *J* = 7.1, 1.6 Hz, 2H), 7.46 (dd, *J* = 8.2, 7.1 Hz, 2H), 5.31 (s, 4H).

**Preparation of 1,8-bis(dibromomethyl)naphthalene (10)**

To a solution of bis(bromomethyl)naphthalene **9** (2.0 g, 6.4 mmol) in carbon tetrachloride (40 mL), were added *N*-bromosuccinimide (2.3 g, 13 mmol) and azobis(isobutyronitrile) (260 mg, 1.58 mmol), and the mixture was heated under reflux for 1 d. After the addition of water (25 mL), the mixture was extracted with ethyl acetate (30 mL × 3). The organic layer was dried over magnesium sulfate and evaporated, and the residue was treated via column chromatography on silica gel to afford 1,8-bis(dibromomethyl)naphthalene (**10**) (1.65 g, 3.5 mmol, 55%, eluted by hexane/EtOAc = 9/1) as pale-yellow crystals.

Mp. 106–109 °C. ^1^H NMR (400 MHz, CDCl_3_) δ 8.52 (dd, *J* = 7.5, 1.3 Hz, 2H), 7.85 (dd, *J* = 8.1, 1.3 Hz, 2H), 7.66–7.56 (m, 2H), 7.66 (s, 2H); ^13^C NMR (100 MHz, CDCl_3_) δ 137.1 (C), 134.3 (CH), 133.5 (C), 132.0 (CH), 126.1 (CH), 123.5 (C), 40.2 (CH); IR (KBr/cm^−1^) 672; HRMS (ACPI-MS) calcd. for (M + H⁺ − Br) C_12_H_8_Br_3_: 390.8156, found 390.8200.

**Nitration reaction for *peri*-substituted naphthalenes**

To a solution of *peri*-substituted naphthalene (0.3 mmol) in chloroform (3 mL), a solution of nitric acid HNO_3_ (*d* = 1.42, 53 μL, 1.2 mmol) in acetic anhydride (1.5 mL) was added. After stirring the resulting mixture at room temperature for 4 h, water (10 mL) was added, and the mixture was extracted with chloroform (20 mL × 3). The organic layer was washed with 2 M of a sodium hydrogen carbonate aqueous solution (10 mL), dried over magnesium sulfate, and evaporated. The residue was treated via column chromatography on silica gel to afford nitrated products (eluted by hexane/EtOAc = 9/1).

1,8-Dimethyl-2-nitronaphthalene [21]: ^1^H NMR (400 MHz, CDCl_3_) δ 7.74 (*d*, *J* = 8.4 Hz, 1H), 7.71 (br d, *J* = 7.6 Hz, 1H), 7.61 (d, *J* = 8.4 Hz, 1H), 7.45 (dd, *J* = 7.6, 7.6 Hz, 1H), 7.41 (br d, *J* = 7.6 Hz, 1H), 2.94 (s, 3H), 2.88 (s, 3H). 

1,8-Dimethyl-4-nitronaphthalene [22]: ^1^H NMR (400 MHz, CDCl_3_) δ 8.22 (dd, *J* = 8.8, 1.3 Hz, 1H), 7.88 (d, *J* = 7.1 Hz, 1H), 7.50 (dd, *J* = 8.8, 7.8 Hz, 1H), 7.40 (d, *J* = 7.1 Hz, 1H), 7.31 (dd, *J* = 7.8, 1.3 Hz, 1H), 2.99 (s, 3H), 2.96 (s, 3H). 

1,8-Bis(bromomethyl)-4-nitronaphthalene: Yellow solid; Mp. 114–117 °C. ^1^H NMR (400 MHz, CDCl_3_) δ 8.30 (dd, *J* = 8.8, 1.4 Hz, 1H), 7.94 (d, *J* = 8.0 Hz, 1H), 7.76 (dd, *J* = 7.2, 1.4 Hz, 1H), 7.69 (dd, *J* = 8.0 Hz, 1H), 7.65 (dd, *J* = 8.8, 7.2 Hz, 1H), 5.26 (s, 4H); ^13^C NMR (100 MHz, CDCl_3_) δ 149.8 (C), 139.3 (C), 134.5 (CH), 131.1 (CH), 130.1 (C), 128.7 (CH), 127.4 (C), 125.6 (CH), 124.9 (C), 122.1 (CH), 36.1 (CH_2_), 35.1 (CH_2_); IR (KBr/cm^−1^) 1526, 767; HRMS (ACPI-MS) calcd. for (M + H⁺) C_12_H_9_Br_2_NO_2_: 359.9053, found 359.9072.

**Hydrogenation reaction for *peri*-substituted naphthalenes**

To a solution of *peri*-substituted naphthalene (0.3 mmol) in ethanol (8 mL), 5 wt% Pd/C (10 mg, 5 μmol, 2 mol%) was added. After bubbling hydrogen gas for 1 min, the test tube was sealed and stirred at room temperature for 1 h. After filtration using celite, the filtrate was concentrated, and the residue was subjected to an NMR analysis. 

## 4. Conclusions

The correlation between the bulkiness of the *peri*-substituents and distortion of the naphthalene ring was studied. The X-ray crystallography showed that 1,8-bis(bromomethyl)naphthalene 9 possesses a vertical strain with an 11.0° dihedral angle between the *peri*-substituents, disturbing the coplanarity of the naphthalene ring. On the other hand, the vertical distortion of 1,8-bis(dibromomethyl)naphthalene 10 was smaller (8.3°), even though the substituents became bulkier. In this case, horizontal distortion was also observed in addition to vertical strain, that is, the inner bond distance of C1–C9 is longer, and the outer bond distances (C1–C2, C2–C3, and C4–C10) are shorter than those of the other derivatives. The atom distances (C1–C8 and C2-C9) of 10 are longer than those of 7–9, and the distance between C4 and C5 is shorter than in other substrates.

The distorted naphthalene ring was expected to show a higher level of reactivity because of its decreased aromaticity. To confirm this, we studied two reactions, nitration and hydrogenation, using 7–10. However, a systematic evaluation method for non-electronic activation was not established because the inductive electron-withdrawing and steric effects were still influenced. Although it is necessary to further evaluate the reactivity, the correlation between the bulkiness of the *peri*-substituents and the distortion of the naphthalene ring will be useful information for researchers who study the physical properties of aromatic compounds and their modifications.

## Figures and Tables

**Table 1:** Selected crystallographic information for crystal structures **7**–**10**.

	7 (R = CH_3_)	8 (R = CH_2_OH)	9 (R = CH_2_Br)	10 (R = CHBr_2_)
Empirical formula	C_12_H_12_	C_12_H_12_O_2_	C_12_H_10_Br_2_	C_12_H_8_Br_4_
Crystal system	Monoclinic	Monoclinic	Monoclinic	Monoclinic
Space group	*P*2_1_/*n*	*P*2_1_/*n*	*C*2/*c*	*P*2_1_/*n*
*ɑ* (Å)	9.5783(6)	8.4875(4)	23.3250(11)	10.1755(4)
*b* (Å)	6.8687(4)	4.8113(2)	7.5802(3)	6.7237(2)
*c* (Å)	13.2814(10)	22.4730(9)	12.3748(6)	19.3037(8)
*β* (°)	92.066(7)	94.379(4)	95.479(4)	100.207(4)
Z	4	4	8	4
Goodness-of-fit	1.051	1.098	1.097	1.031
R_1_ [*I* > 2*σ*(*I*)] wR_2_ (all data)	*R*_1_ = 0.054, *wR*_2_ = 0.160	*R*_1_ = 0.041, *wR*_2_ = 0.110	*R*_1_ = 0.036, *wR*_2_ = 0.107	*R*_1_ = 0.048, *wR*_2_ = 0.132
CCDC number	2266182	2266183	2266184	2266185

**Table 2:** Comparison of dihedral angles α (between C1–C11 and C8–C12 bonds), β (between C1–C9 and C4–C10 bonds), and γ (between C1–C9 and C5–C10 bonds) in naphthalenes **7**–**10**.

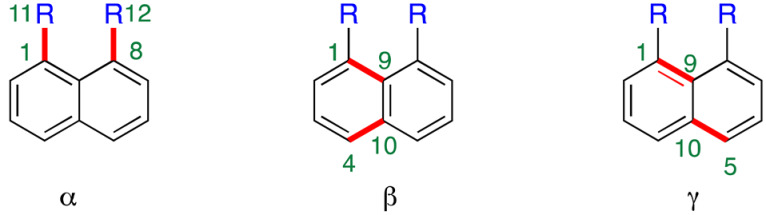				
R		α/°	β/°	γ/°
CH_3_	**7**	0.7	0.5	0.1
CH_2_OH	**8**	4.6	2.1	1.5
CH_2_Br	**9**	11.0	4.1	4.9
CHBr_2_	**10**	8.3	3.5	3.1

**Table 3:** Bond angles around the C9 and C10 carbons in **7–10**.

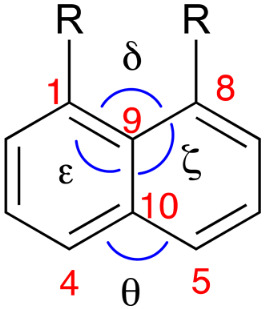					
R		δ/°	ε/°	ζ/°	θ/°
CH_3_	**7**	126.3(1)	116.6(9)	117.1(9)	118.2(1)
CH_2_OH	**8**	126.3(2)	117.1(2)	116.7(2)	118.5(3)
CH_2_Br	**9**	128.4(6)	115.1(5)	116.5(5)	117.6(2)
CHBr_2_	**10**	125.5(1)	117.2(1)	117.3(1)	118.8(2)

**Table 4:** Bond lengths (Å) in the naphthalene rings in **7**–**10**.

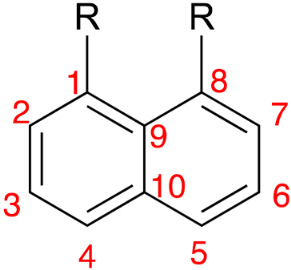				
	7 (R = CH_3_)	8 (R = CH_2_OH)	9 (R = CH_2_Br)	10 (R = CHBr_2_)
C1–C2	1.375(2)	1.380(2)	1.380(4)	1.37(1)
C2–C3	1.407(2)	1.407(2)	1.404(4)	1.38(1)
C3–C4	1.358(2)	1.364(2)	1.358(4)	1.37(1)
C4–C10	1.419(2)	1.421(2)	1.423(4)	1.407(9)
C10–C9	1.442(2)	1.435(1)	1.430(4)	1.434(9)
C9–C1	1.445(2)	1.447(2)	1.446(4)	1.481(9)
C10–C5	1.418(2)	1.422(2)	1.422(4)	1.414(9)
C5–C6	1.358(2)	1.361(2)	1.360(5)	1.34(1)
C6–C7	1.409(2)	1.407(2)	1.400(5)	1.41(1)
C7–C8	1.377(2)	1.380(2)	1.382(4)	1.37(1)
C8–C9	1.444(2)	1.446(2)	1.446(4)	1.434(9)

Values smaller than others are colored with blue, and values larger than others are colored with red.

**Table 5:** Selected interatom distances (Å) in **7**–**10**.

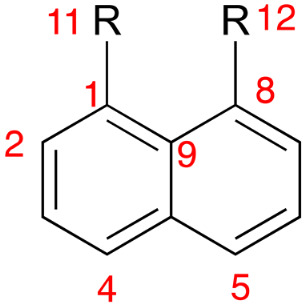				
	7 (R = CH_3_)	8 (R = CH_2_OH)	9 (R = CH_2_Br)	10 (R = CHBr_2_)
C1–C8	2.569(2)	2.580(2)	2.580(4)	2.625(9)
C2–C9	2.429(2)	2.444(2)	2.440(4)	2.459(9)
C4–C5	2.442(2)	2.440(2)	2.445(5)	2.413(9)
C11–C12	2.961(2)	2.984(2)	3.058(4)	2.987(9)

Values smaller than others are colored with blue, and values larger than others are colored with red.

**Table 6:** Nitration of 1,8-disubstituted naphthalenes **7**, **9**, and **10**.

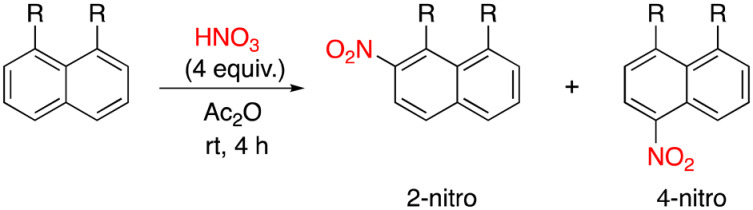					
Entry	Naphthalene		Yield/%		Recovery/%
	R		2-nitro	4-nitro	
1	CH_3_	**7**	44	56	0
2	CH_2_Br	**9**	0	12	62
3	CHBr_2_	**10**	0	0	61

**Table 7:** Comparison of actual and calculated C–C and C–Br bond lengths (Å) in **7**, **9**, and **10**.

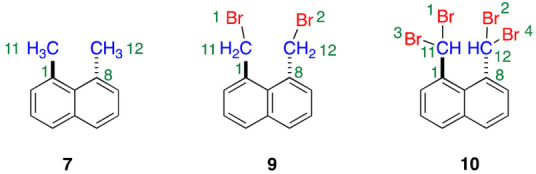								
R			C11–Br1	C11–Br3	C12–Br2	C12–Br4	C1–C11	C8–C12
CH3	**7**	Crystal	—	—	—	—	1.507(2)	1.513(2)
		calcd.	—	—	—	—	1.517	1.517
CH2Br	**9**	Crystal	1.981(3)	—	1.996(3)	—	1.499(4)	1.489(4)
		calcd.	2.020	—	2.021	—	1.496	1.496
CHBr2	**10**	Crystal	1.986(8)	1.956(7)	1.977(7)	1.947(8)	1.51(1)	1.49(1)
		calcd.	1.996	1.976	1.996	1.976	1.502	1.502

**Table 8:** Homo and LUMO levels (eV) of **7**, **9**, and **10**.

R		HOMO	LUMO
CH3	**7**	−5.51	−0.80
CH2Br	**9**	−6.11	−1.83
CHBr2	**10**	−6.29	−2.19

**Table 9:** NICS calculations for **7**, **9**, and **10**.

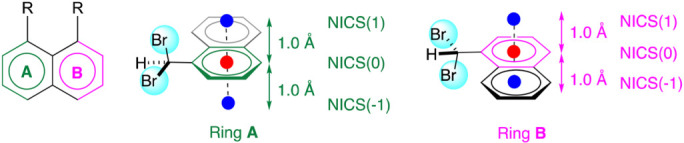							
R		Ring A			Ring B		
		NICS(−1)	NICS(0)	NICS(1)	NICS(−1)	NICS(0)	NICS(1)
CH3	**7**	−11.1	−9.0	−11.1	−11.1	−9.0	−11.1
CH2Br	**9**	−11.6	−9.9	−11.1	−11.1	−9.9	−11.6
CHBr2	**10**	−11.7	−9.3	−10.7	−10.6	−9.3	−11.7

## Data Availability

Not applicable.

## Supplementary Materials

The following supporting information can be downloaded at: https://www.mdpi.com/article/10.3390/molecules28145343/s1. ^1^H and ^1^H-^1^H NOESY 2D NMR spectra of 7–10. ^13^C NMR and ACPI-MS spectra of 10. ^1^H and ^13^C NMR spectra and ACPI-MS spectrum of nitrated compounds. Chemical shifts of 1,8-disubstituted naphthalenes **7**–**10**.
