# Supplementary material for: A Study of the Correlation between the Bulkiness of peri-Substituents and the Distortion of a Naphthalene Ring
Source: Molecules. 2023 Jul 11;28(14):5343. doi: 10.3390/molecules28145343 (PMC10386483; doi:10.3390/molecules28145343)
Supplement: Supplementary file 1 [file molecules-28-05343-s001.zip › molecules-2475530-supplementary.pdf]

Supplementary Materials

1,8-Dimethylnaphthalene (7)

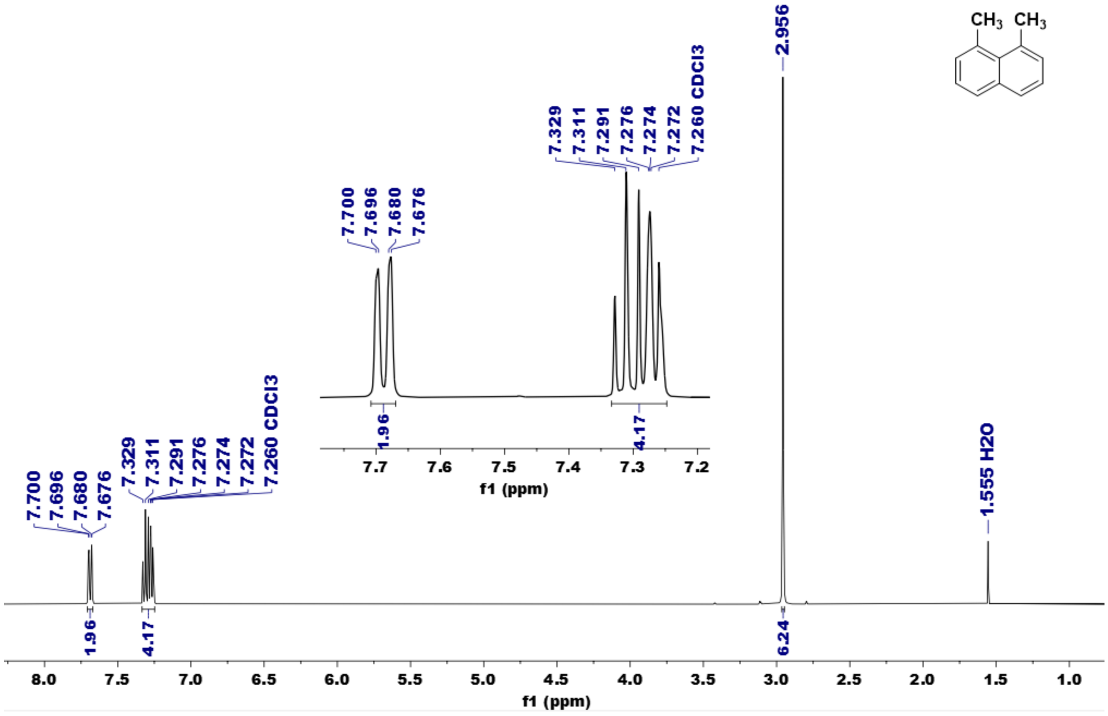

^{1}H NMR ($CDCl_3$, 400 MHz) of compound **7**

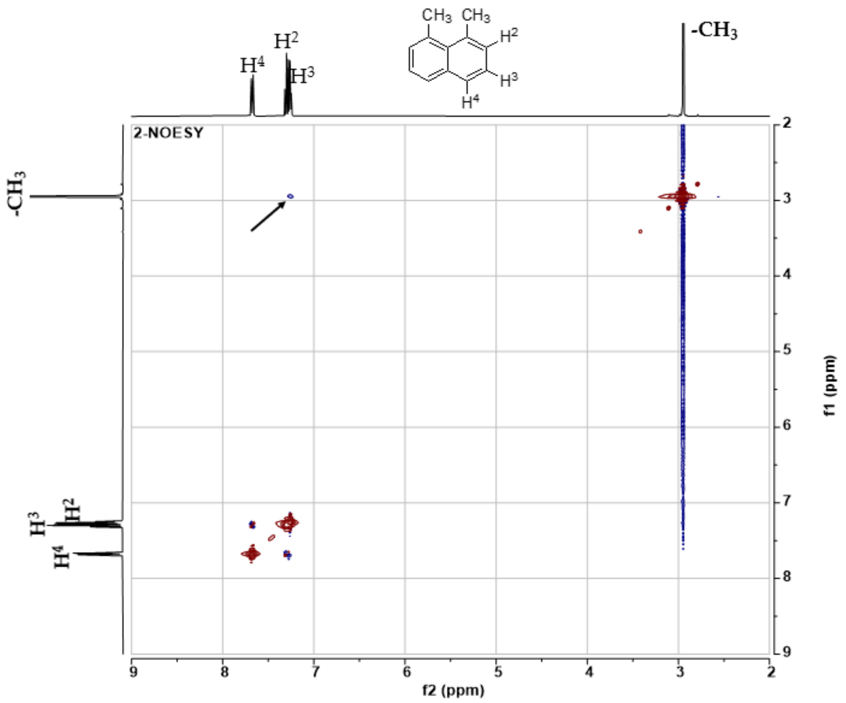

NOESY spectrum of compound **7**

1,8-Bis(hydroxymethyl)naphthalene (8)

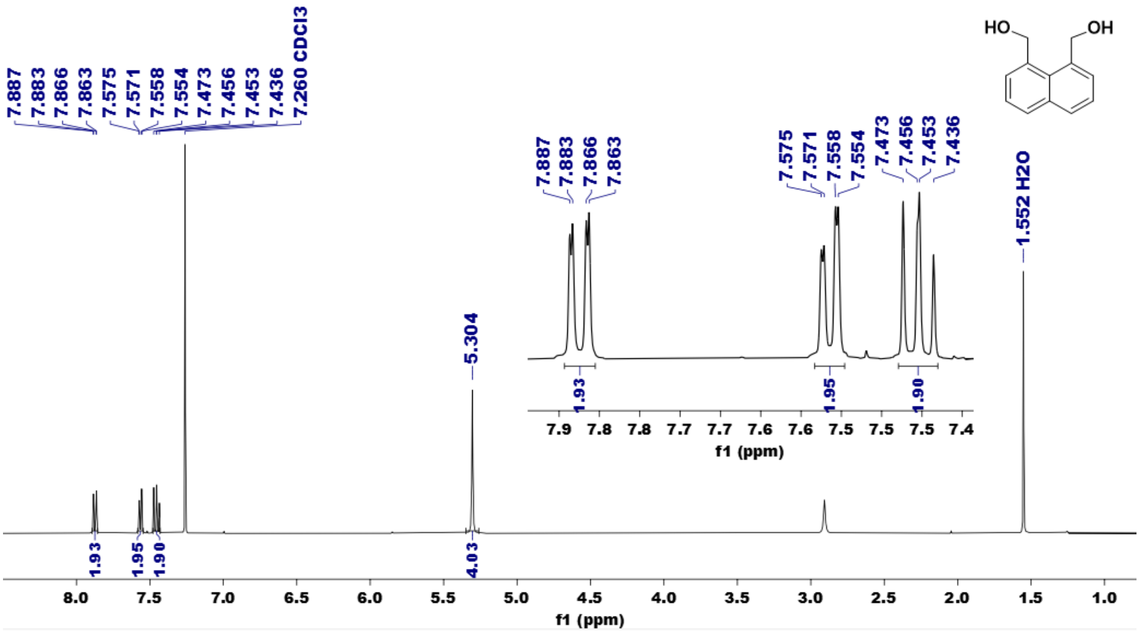

^{1}H NMR ($CDCl_3$, 400 MHz) of compound **8**

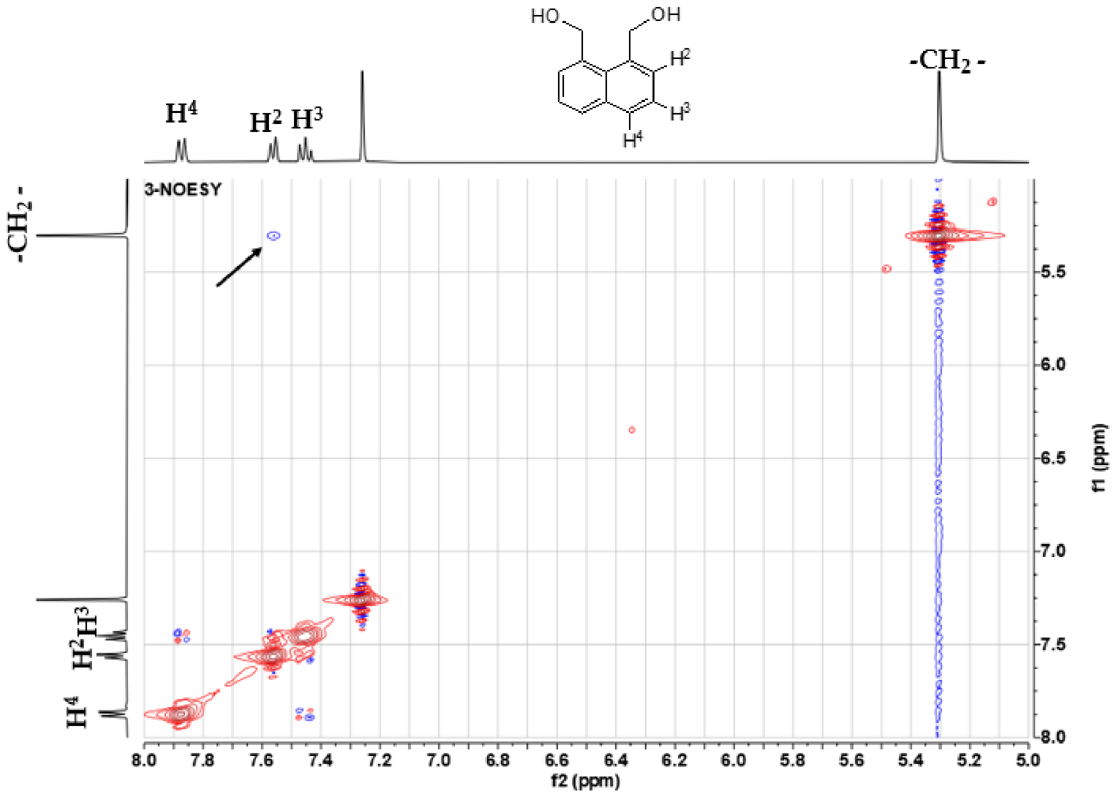

NOESY spectrum of compound **8**

1,8-Bis(bromomethyl)naphthalene (9)

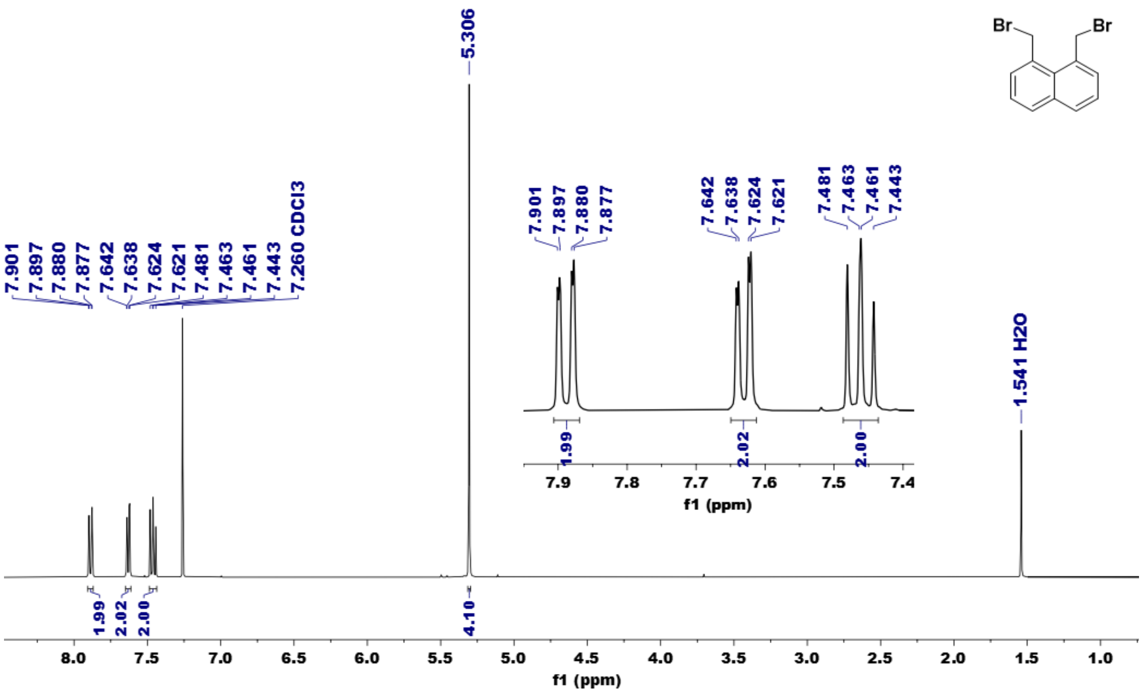

^{1}H NMR ($CDCl_3$, 400 MHz) of compound **9**

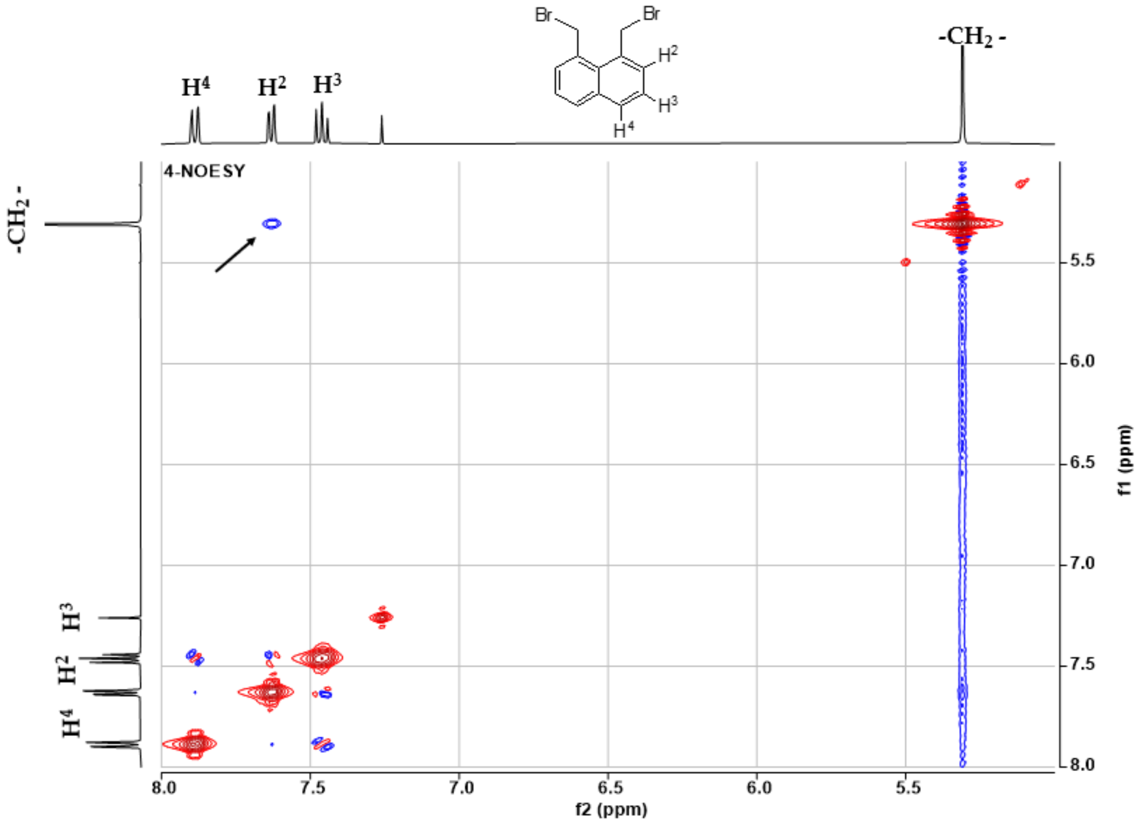

NOESY spectrum of compound **9**

1,8-Bis(dibromomethyl)naphthalene (10)

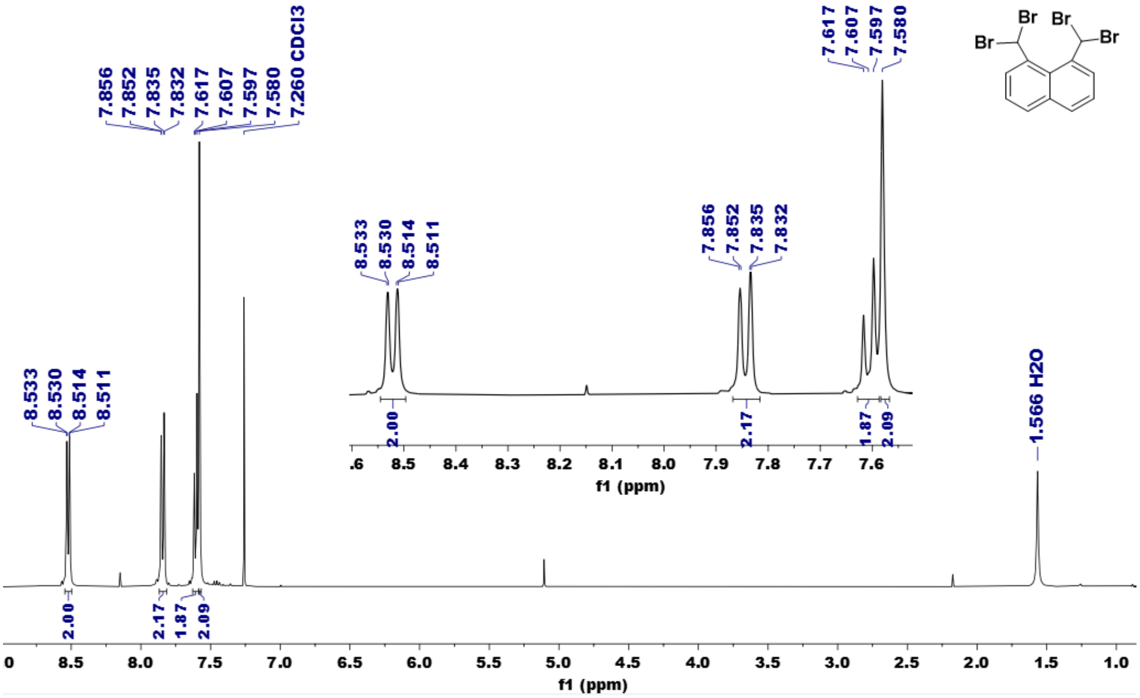

^{1}H NMR ($CDCl_3$, 400 MHz) of compound **10**

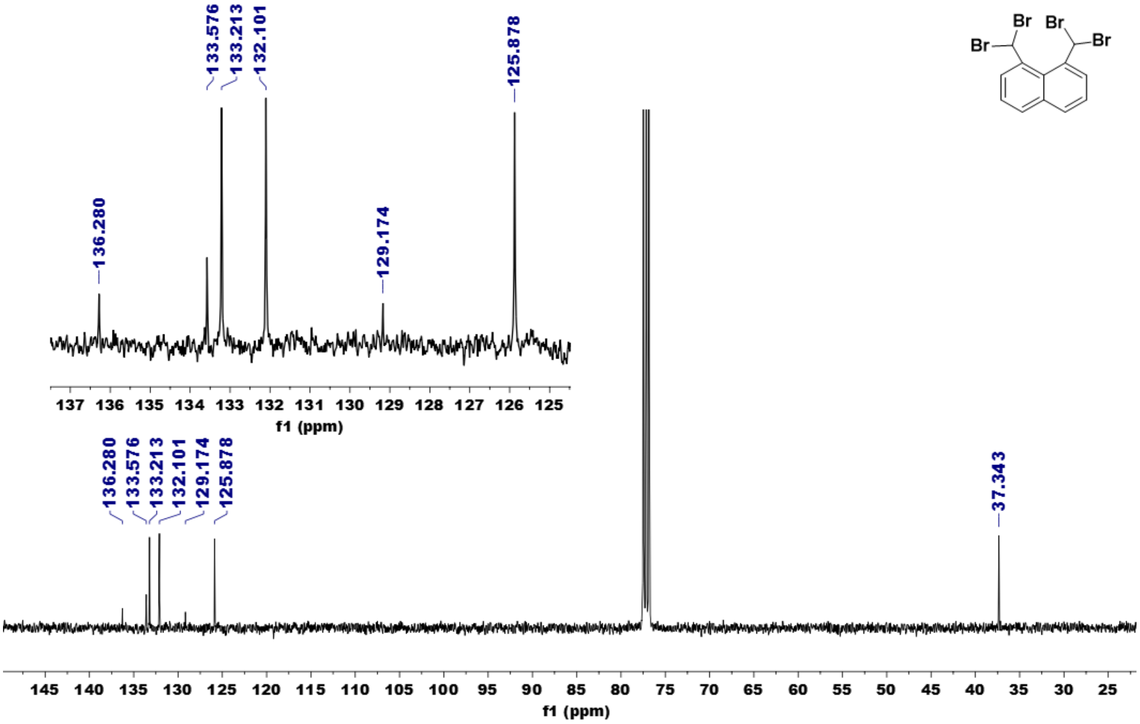

^{13}C NMR ($CDCl_3$, 100 MHz) of compound **10**

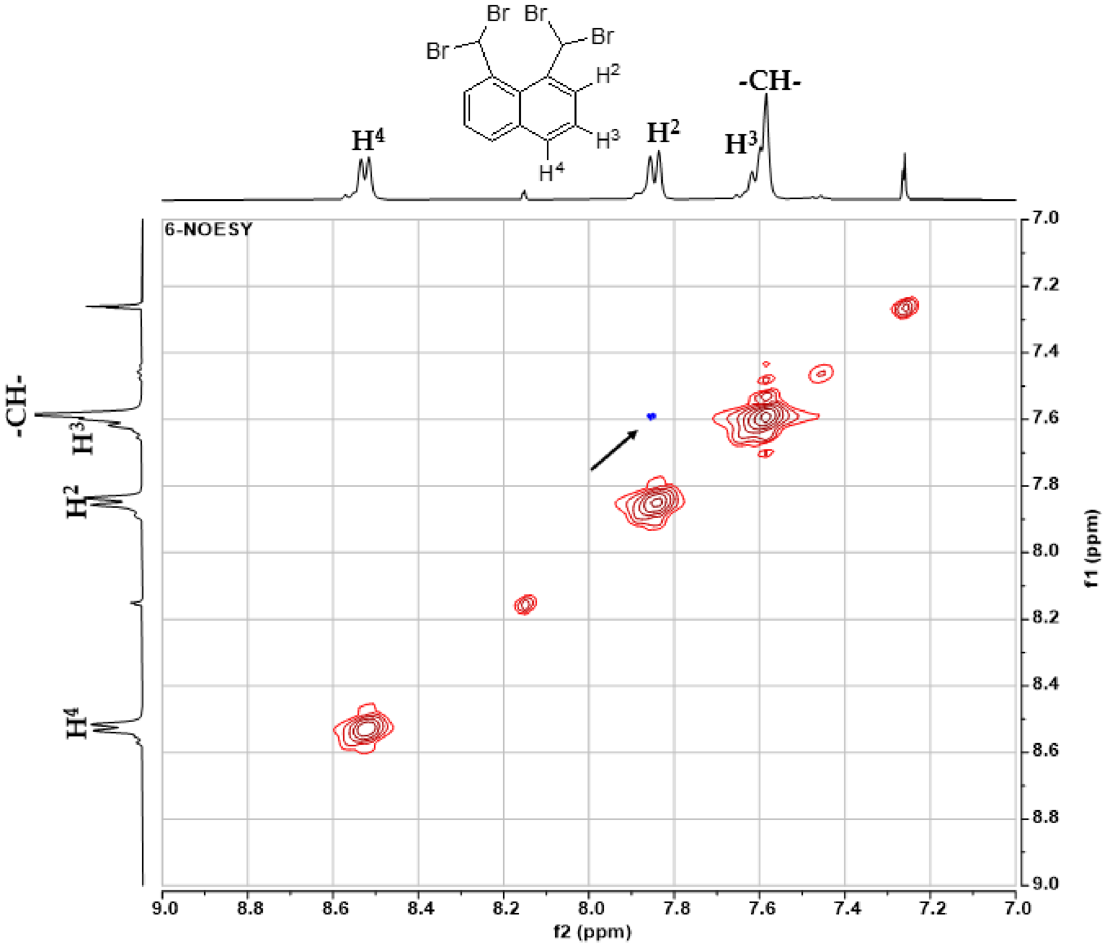

NOESY spectrum of compound **10**

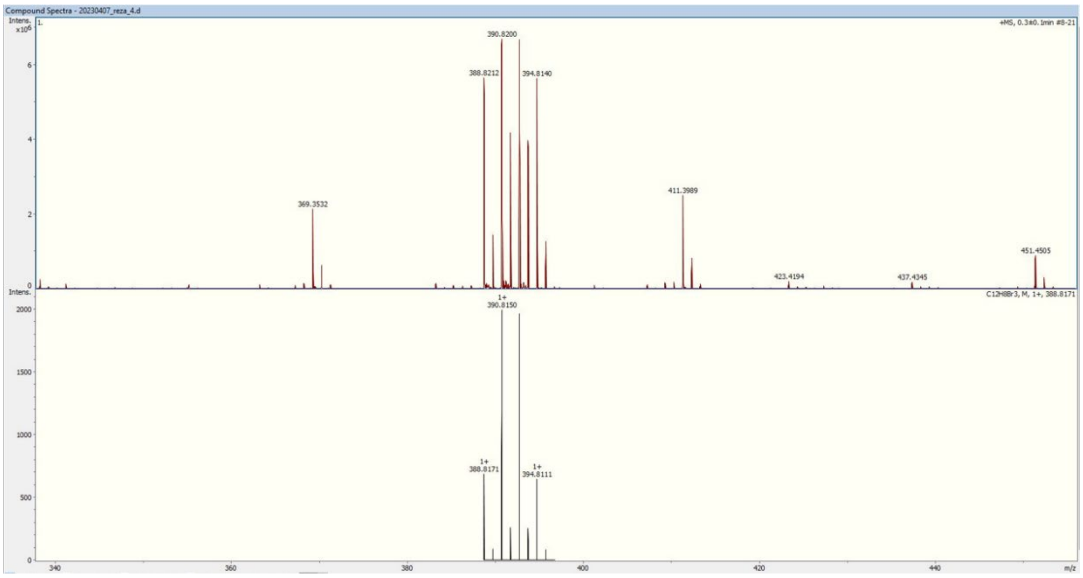

ACPI-MS spectrum of compound **10**

1,8-Dimethyl-2-nitronaphthalene

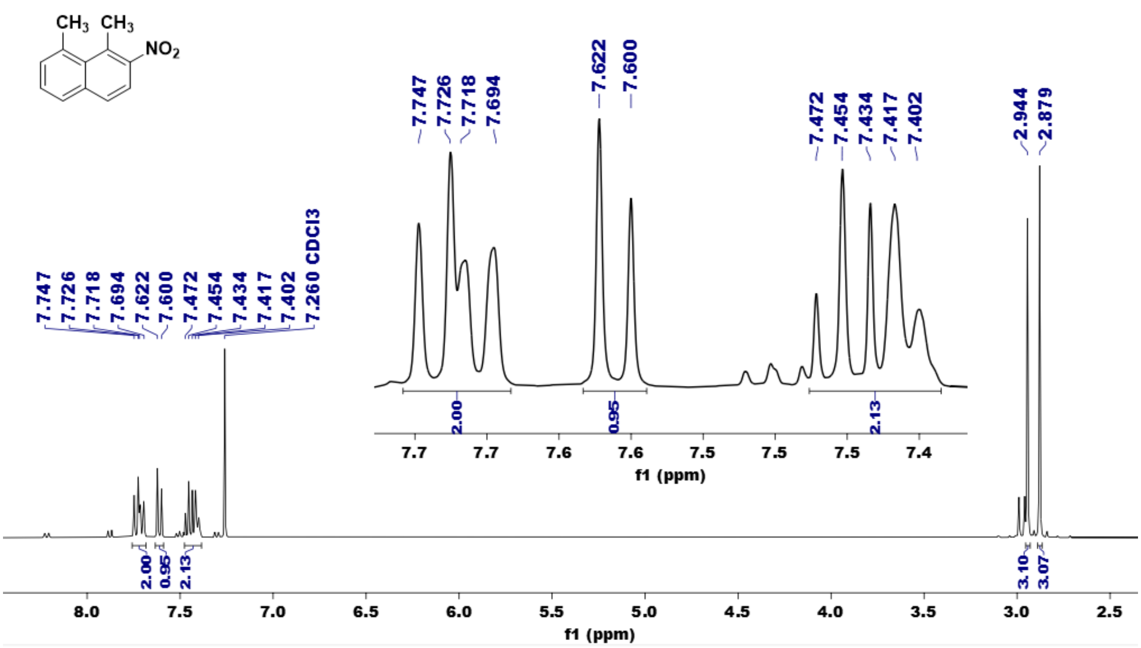

^1H NMR ($CDCl_3$, 400 MHz)

1,8-Dimethyl-4-nitronaphthalene

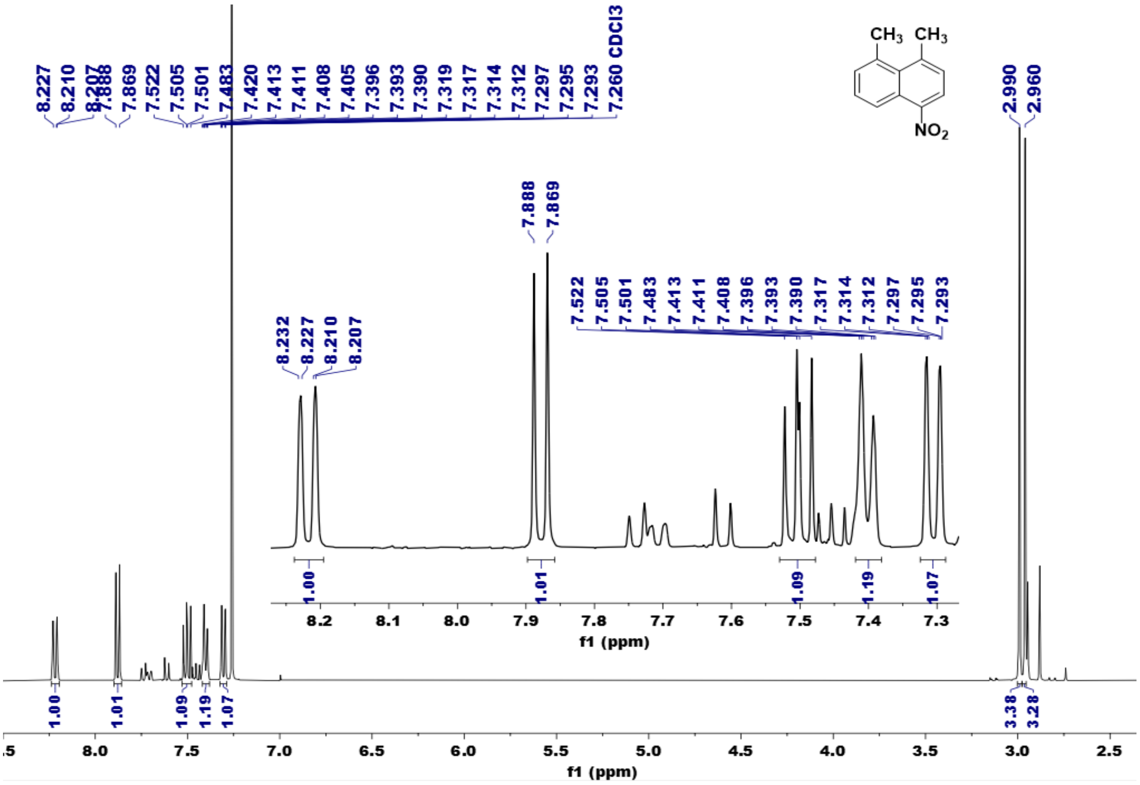

^1H NMR ($CDCl_3$, 400 MHz)

1,8-(Bisbromomethyl)-4-nitronaphthalene

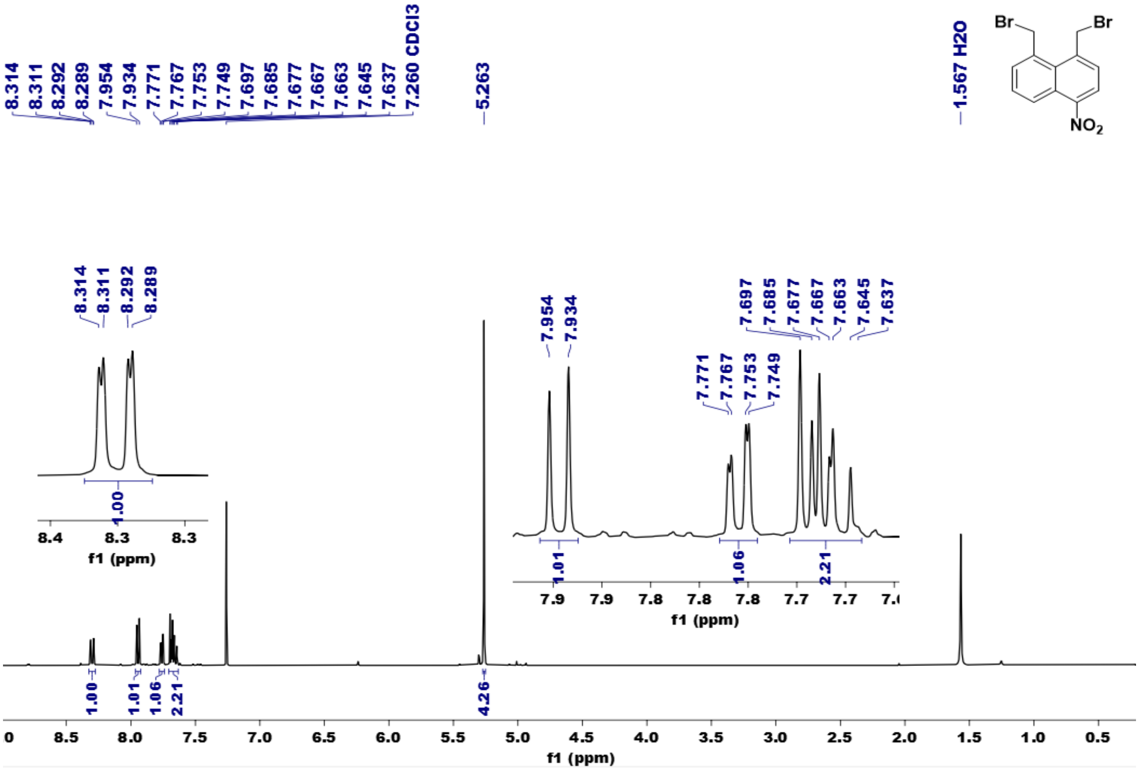

^{1}H NMR ($CDCl_3$, 400 MHz)

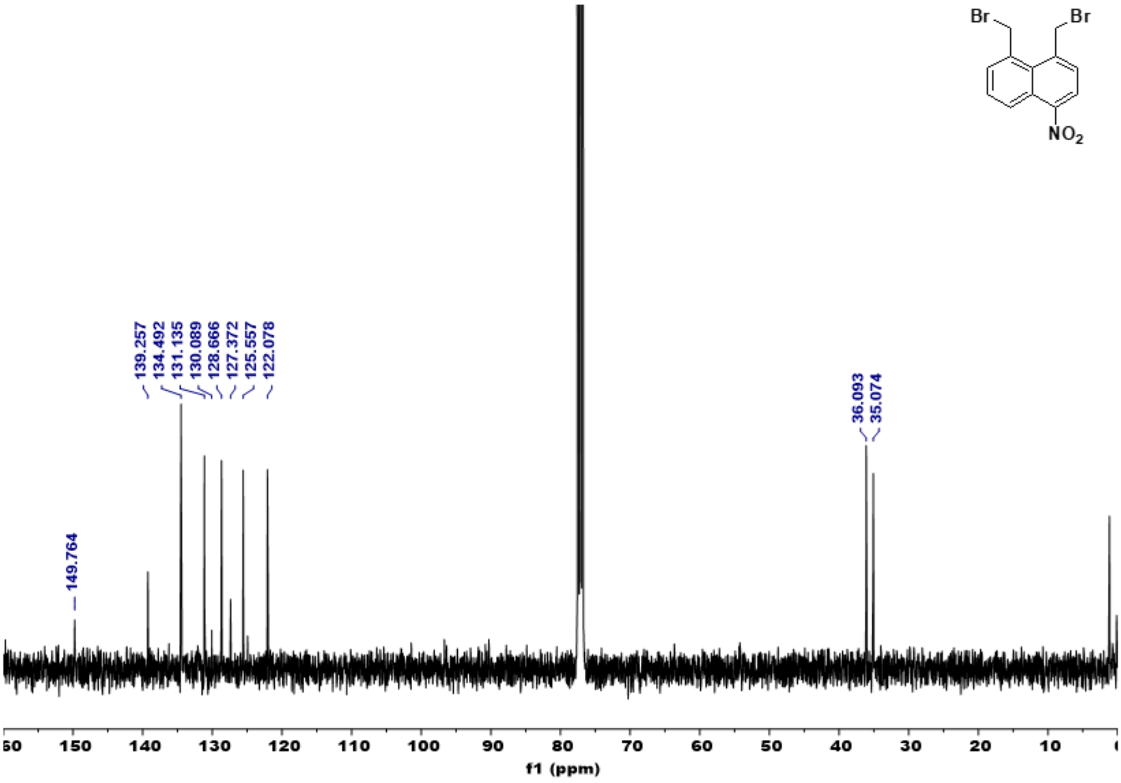

^{13}C NMR ($CDCl_3$, 100 MHz) of compound

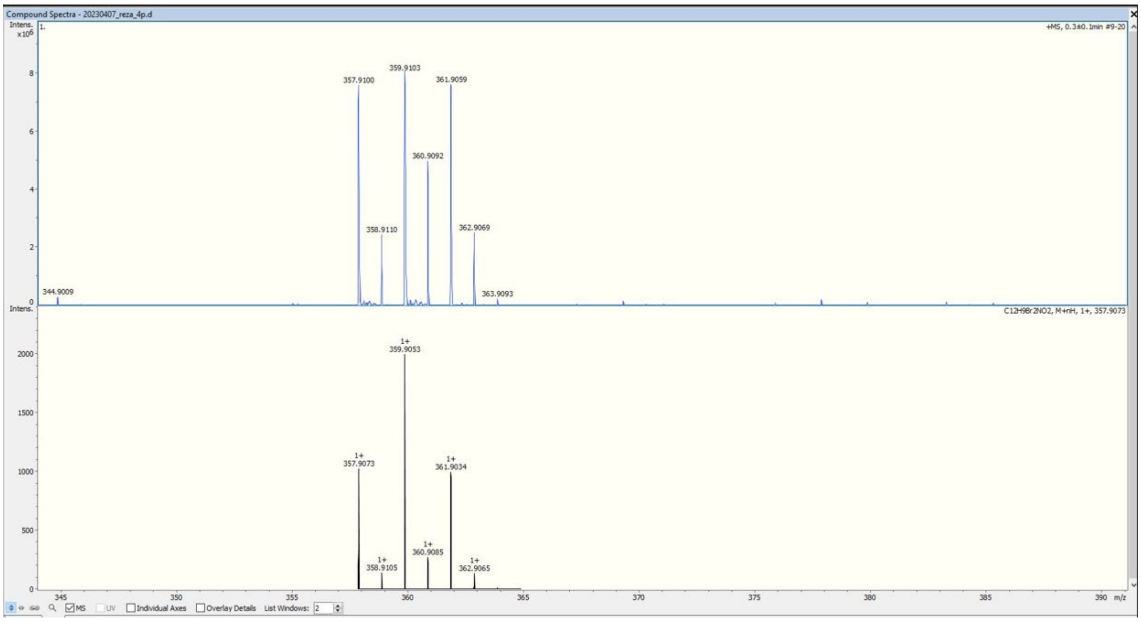

ACPI-MS spectrum of compound

NMR spectra of 1,8-disubstituted naphthalenes **7–10**

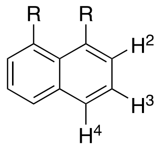

Naphthalene		Chemical shift/ppm		
R		H^2	H^3	H^4
CH_3	**7**	7.31	7.23	7.67
CH_2OH	**8**	7.56	7.45	7.87
CH_2Br	**9**	7.63	7.46	7.89
$CHBr_2$	**10**	7.85	7.66	8.52
